# Esculetin Ameliorates Psoriasis-Like Skin Disease in Mice by Inducing CD4^+^Foxp3^+^ Regulatory T Cells

**DOI:** 10.3389/fimmu.2018.02092

**Published:** 2018-09-12

**Authors:** Yuchao Chen, Qunfang Zhang, Huazhen Liu, Chuanjian Lu, Chun-Ling Liang, Feifei Qiu, Ling Han, Zhenhua Dai

**Affiliations:** Section of Immunology and Joint Immunology Program, Guangdong Provincial Academy of Chinese Medical Sciences and Guangdong Provincial Hospital of Chinese Medicine, Guangzhou, China

**Keywords:** esculetin, immunosuppression, immunoregulation, psoriasis, regulatory T cell

## Abstract

Psoriasis is an autoimmune and inflammatory skin disease affecting around 2–3% of the world's population. Patients with psoriasis need extensive treatments with global immunosuppressive agents that may cause severe side effects. Esculetin, a type of coumarins, is an active ingredient extracted mainly from the bark of Fraxinus rhynchophylla, which has been used to treat inflammatory and autoimmune diseases in China. However, the antipsoriatic effects of esculetin have not been reported. In this study, we aimed to investigate the effects of esculetin on psoriatic skin inflammation in a mouse model and explored the potential molecular mechanisms underlying its action. We found that esculetin ameliorated the skin lesion and reduced PASI scores as well as weight loss in imiquimod-induced psoriasis-like mice, accompanied with weakened proliferation and differentiation of keratinocytes and T cell infiltration in esculetin-treated psoriatic mice. In addition, esculetin reduced the frequency of CD8^+^CD44^high^CD62L^low^ effector T cells in psoriatic mice. In contrast, it increased the frequency of CD4^+^Foxp3^+^ Tregs in both lymph nodes and spleens of the psoriatic mice while promoting the differentiation of CD4^+^CD25^−^ T cells into CD4^+^Foxp3^+^ Tregs *in vitro*. Interestingly, depleting CD4^+^Foxp3^+^ Tregs largely reversed esculetin-mediated reduction in PASI scores, indicating that esculetin attenuates murine psoriasis mainly by inducing CD4^+^Foxp3^+^ Tregs. Furthermore, the mRNA levels of proinflammatory cytokines in the psoriatic mouse skin, including IL-6, IL-17A, IL-22, IL-23, TNF-α, and IFN-γ, were dramatically decreased by the treatment with esculetin. Finally, we found that esculetin inhibited the phosphorylation of IKKα and P65 in the psoriatic skin, suggesting that it inhibits the activation of NF-κB signaling. Thus, we have demonstrated that esculetin attenuates psoriasis-like skin lesion in mice and may be a potential therapeutic candidate for the treatment of psoriasis in clinic.

## Introduction

Psoriasis is an inflammatory skin disease, which affects about 2–3% of the world's population (1, 2). Psoriatic lesions are characterized by parakeratosis, epidermal hyperplasia, and the inflammatory immune cell infiltration in the dermis (3). Meanwhile, patients with psoriasis may have an increased risk of comorbidities, such as arthritis, metabolic syndrome, cardiovascular disease, obesity, and even cancer (4–8). Although the exact pathogenesis of psoriasis remains unclear, it is widely accepted that aberrant activation of immune cells and keratinocytes in the dermis is responsible for the immunopathology of psoriasis (9). In particular, NF-κB signaling pathway in keratinocytes is activated by the cytokines released from immune cells, resulting in the proliferation of epidermal keratinocytes (10). Psoriatic patients are generally treated with glucocorticosteroids and/or conventional immunosuppressants. Although these drugs can alleviate psoriatic skin lesion in the short term, they may cause serious side effects, including hepatotoxicity, nephrotoxicity, infection, and even cancer (11–13). Therefore, it is necessary to seek a new drug, especially a natural product with less adverse effects for the treatment of psoriasis.

Esculetin, 6, 7-dihydroxycoumarin, is an active ingredient originally isolated from the bark of some natural plants, including *Fraxinus rhynchophylla* Hance, *Fraxinus chinensis* Roxb, and *Fraxinus aboana* Lingelsh (14, 15). It has been shown that esculetin has some biological and pharmacological activities, including anti-oxidative (16, 17), anti-inflammatory (18), anti-viral (19), anti-asthmatic (20), and anti-tumor effects (21, 22). As an anti-inflammatory drug, esculetin can exert therapeutic effects on several inflammatory diseases, such as hepatic fibrosis (23), arthritis (24), and atopic dermatitis (25). Previous studies have also reported that esculetin inhibits T cell activation without suppressing IL-2 production or IL-2 receptor expression (26). In addition, esculetin can protect mice from acute lung injury and inhibit lung cancer growth by downregulating NF-κB activation (27, 28). Interestingly, esculetin can also ameliorate atopic dermatitis via suppressing NF-κB activation (25). However, it remains unknown if esculetin can treat psoriasis. The upregulation of NF-κB activation in psoriasis-like mice (29) and psoriatic patients (30) has previously been demonstrated. Thus, esculetin could treat psoriasis by suppressing NF-κB activation.

In this study, we investigated the therapeutic effects of esculetin on imiquimod (IMQ)-induced psoriasis in mouse models. We found that esculetin significantly ameliorated psoriatic skin lesion, reduced PASI scores, improved skin immunopathology and hindered T cell infiltration in IMQ-induced psoriasis-like mice. Esculetin also significantly increased the frequency of CD4^+^Foxp3^+^ Tregs while reducing the frequency of CD8^+^ effector T cells. Furthermore, esculetin inhibited the expression of important proinflammatory cytokines and downregulated NF-κB signaling.

## Material and methods

### Materials and animals

Imiquimod cream (5%) was obtained from Sichuan Mingxin Pharmaceutical Co., Ltd. (Sichuan, China). Esculetin (Mw 178.14, purity > 99%) was obtained from Chengdu Push Bio-technology Co., Ltd. (Chengdu, China). Purified anti-CD3 and anti-CD28 monoclonal Abs were purchased from eBioscience (San Diego, CA, United States). Anti-CD25 depleting antibody (PC61) was also purchased from eBioscience.

BALB/c mice (6–8 week-old, 20 ± 2 g) were purchased from Guangdong Medical laboratory Animal Center (Guangzhou, China). All mice were housed under a pathogen-free condition and provided free access to food and water. All animal experiments were performed according to the Chinese National Guidelines for the Care and Use of Laboratory Animals and approved by the Animal Experimental Ethics Committee of Guangdong Provincial Hospital of Chinese Medicine.

### Imiquimod-induced psoriasis-like mouse model and animal treatment

BALB/c mice were randomly divided into four groups, including control group, vehicle group, Esculetin low-dose group (ES-L) and Esculetin high-dose group (ES-H). The back hair of all the mice was shaved in an area of 3 × 2 cm. To establish a mouse model of psoriasis, all groups except the control group were topically administered with a dose of 62.5 mg imiquimod cream (containing 5% imiquimod) on the back skin of mice for 7 consecutive days. Esculetin was prepared by 10% PEG400 solution. The mice of ES-L and ES-H groups were orally administered with esculetin at a dose of 50 and 100 mg/kg/day, respectively, for 7 consecutive days. While the control group and vehicle group were given 10% PEG400 solution daily for 7 consecutive days, all of the treatments began from the day when IMQ was administered. After 7 days, mice were sacrificed and their skin, spleens and lymph nodes were collected for further analyses. To deplete CD4^+^Foxp3^+^ Tregs, mice in some groups were treated *i.p*. with anti-CD25 mAb (Clone: PC61, eBioscience) at 0.2 mg on days 0, 2, 4, and 6 after IMQ was applied. This treatment with PC61 generally depletes around 80% of the CD4^+^CD25^+^ Tregs, as described in our previous studies (31).

### Scoring the severity of psoriatic skin lesion

The severity of skin lesion was graded by Psoriasis Area and Severity Index (PASI), which comprises the parameters of skin erythema, scaling, and thickness. PASI was scored on a scale from 0 to 4. “0” represents none; “1” represents “slight”; “2” represents “moderate”; “3” represents “marked”; “4” represents “severe”. The mice of each group were scored from the day IMQ was administered for seven consecutive days. The scores were used for evaluating the severity of psoriatic skin lesion.

### Histological analysis and immunohistochemistry

Skin samples from the mice were fixed in 4% neutral paraformaldehyde for 24 h and then embedded in paraffin. The samples in paraffin were cut into 3 μm-thick sections and placed on slides. The slides were then used for H&E and immunohistochemistry staining. For immunohistochemistry staining, slides were incubated with primary monoclonal anti-CD3, anti-CD8, or anti-Ki67 antibody at a dilution of 1:100 (Abcam) at 4°C overnight, then secondary antibody HPR-anti-Rabbit IgG (Maxim). The slides were colored with 3′-diaminobenzidene (DAB, Sigma-Aldrich) and counterstained by hematoxylin. For quantitative analysis, slides were imaged at a magnification of 100x. The integrated optical density (IOD) of CD3 in the image was measured using Image Pro plus 6 software.

### Flow cytometric analysis

Draining lymph nodes and spleen cells were harvested and stained for surface markers with anti-CD4-FITC or, separately, anti-CD8-FITC, anti-CD44-V450, and anti-CD62L-APC antibodies (BD Biosciences). To determine intracellular Foxp3 expression, cells were fixed and permeated by Foxp3/Transcription Factor Fixation/Permeabilization kits (eBioscience), and then stained for intracellular FoxP3 with anti-Foxp3-APC monoclonal antibody (eBioscience). The frequencies of CD4^+^Foxp3^+^ Tregs and effector CD8^+^ T cells were finally analyzed through FACSCalibur (BD Biosciences).

### Treg differentiation *in vitro*

CD4^+^CD25^−^ T cells were isolated from naïve BALB/c mice by cell sorting using FACSAria III (BD Biosciences). The isolated cell purity was typically over 98%. Subsequently, cells were cultured in 96-well plates at 5 × 10^5^ cells per well with anti-CD3 (5 μg/ml) plus anti-CD28 mAb (2.5 μg/ml) in the presence or absence of IL-2 (10 ng/ml) plus esculetin (10 μM, 0.1%DMSO) or TGF-β1 (5 ng/ml) for 4 days. The frequency of CD4^+^Foxp3^+^ Tregs was determined via flow analysis.

### Quantitative real-time reverse transcription PCR (RT-PCR)

Total mRNA was extracted from skin tissues with TRIzol reagents (Invitrogen, USA), and mRNA was then transcribed to cDNA with PrimeScript™ RT reagent kit (TAKARA Bio Incorporation, Kusatsu, Japan). The relative mRNA expression levels of cytokines were determined using an ABI 7500 Fast Real-Time PCR System (Thermo Fisher Scientific, USA). GAPDH gene was used as an internal standard gene and the 2^−ΔΔ*CT*^ method was utilized to quantitatively analyze the data. The primer sequences are listed in Table 1.

**Table 1:** Primer sequences of target genes.

**Target gene**		**Primer sequence (5′ → 3′)**
IL-6	Forward	TAGTCCTTCCTACCCCAATTTCC
	Reverse	TTGGTCCTTAGCCACTCCTTC
IL-10	Forward	GCTCTTACTGACTGGCATGAG
	Reverse	CGCAGCTCTAGGAGCATGTG
IL-17A	Forward	TTTAACTCCCTTGGCGCAAAA
	Reverse	CTTTCCCTCCGCATTGACAC
IL-22	Forward	ATGAGTTTTTCCCTTATGGGGAC
	Reverse	GCTGGAAGTTGGACACCTCAA
IL-23	Forward	ATGCTGGATTGCAGAGCAGTA
	Reverse	ACGGGGCACATTATTTTTAGTCT
Foxp3	Forward	CCCATCCCCAGGAGTCTTG
	Reverse	CCCATCCCCAGGAGTCTTG
TNF-α	Forward	ACTGATGAGAGGGAGGCCAT
	Reverse	CCGTGGGTTGGACAGATGAA
IFN-γ	Forward	ATGAACGCTACACACTGCATC
	Reverse	ATGAACGCTACACACTGCATC
K10	Forward	GCCTCCTACATGGACAAAGTC
	Reverse	GCTTCTCGTACCACTCCTTGA
Involucrin	Forward	ATGTCCCATCAACACACACTG
	Reverse	TGGAGTTGGTTGCTTTGCTTG
β-actin	Forward	GGCTGTATTCCCCTCCATCG
	Reverse	CCAGTTGGTAACAATGCCATGT

### Western blotting

Total protein samples derived from skin tissues were obtained with RIPA lyzing buffer followed by centrifugation at 4°C for 10 min at 12,000 rpm. The concentration of proteins in supernatants was measured using a BCA protein assay kit (Thermo Fisher Scientific). Then protein samples were run in 10% SDS-PAGE gel and electro-transferred to a PVDF membrane. The membranes were blocked with TBST containing 5% (w/v) BSA at room temperature for 1 h and subsequently incubated with primary anti-phospho-p65, anti-p65, anti-phospho-IKKα, and anti-IKKα antibodies (1:1,000 or 1:2,000, Cell Signaling Technology, Boston, USA) at 4°C overnight. After incubation, the membranes were washed using TBST and incubated with a secondary antibody, HPR-conjugated goat anti-rabbit or anti-mouse IgG (1:2,000), at room temperature for 1 h. Finally, blots were detected by a Bio-Rad Gel imaging system and analyzed by Image J software.

### Statistical analyses

Data were presented as the mean ± SD and analyzed using GraphPad Prism 6 (GraphPad Software, La Jolla, CA, USA). Statistical comparisons between groups were performed using Student's *t*-test and one-way ANOVA. A value of *P* < 0.05 was considered statistically significant.

## Results

### Esculetin ameliorates skin lesion in IMQ-induced psoriatic mice

**Figure 1:**
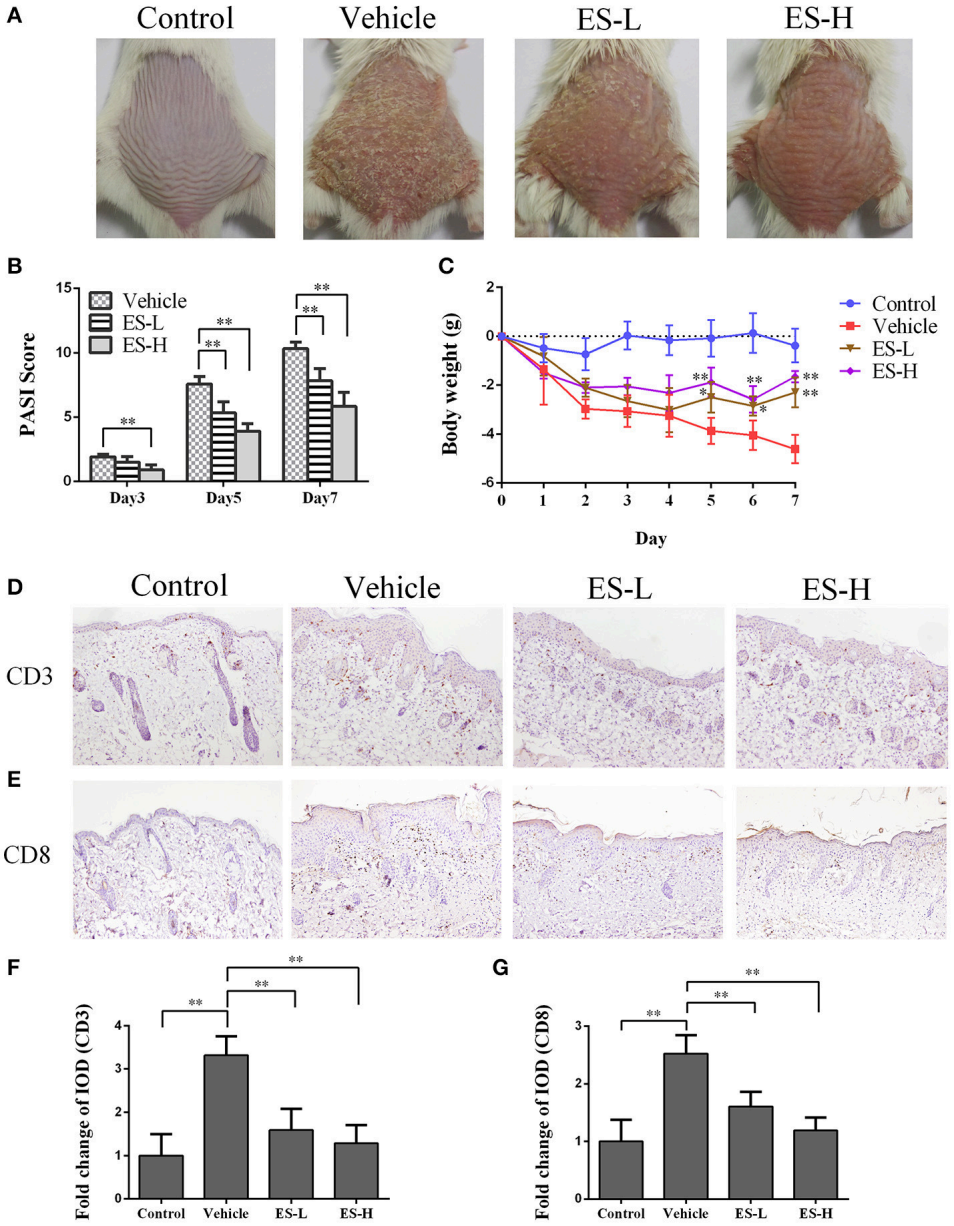
Esculetin reduces the PASI score and ameliorates the skin lesion of IMQ-induced psoriasis-like mice. **(A)** The representatives of photos of dorsal skin in IMQ-induced psoriasis-like mice 7 days after IMQ-treatment without or with esculetin. **(B)** The PASI scores of the skin lesion in IMQ-induced psoriasis-like mice on days 3, 5, and 7 after related treatment. **(C)** The daily mouse weight during the treatment. **(D–G)** Immunohistochemical images of CD3 or CD8 staining (magnification: 100X) of dorsal skin in control or psoriatic mice 7 days after the treatment. Data are presented as the mean ± SD (*n* = 4–6 mice/group, *p < 0.05 and **p < 0.01 vs. vehicle group). One representative of three separate experiments is shown. (ES-L: low-dose of esculetin and ES-H: high-dose of esculetin).

To evaluate whether esculetin would have an effect on psoriasis, we utilized a model of IMQ-induced psoriasis-like mice that were treated with esculetin. The control mice without esculetin did not show any sign of skin inflammation. The symptoms of psoriasis-like lesions, including skin erythema, scaling, and thickening, were severe in the vehicle group 7 days after IMQ treatment. However, treatments with both low-dose of esculetin (ES-L) and high-dose of esculetin (ES-H) reduced erythema, scaling and thickening, and overall skin lesion (Figure 1A). The PASI scores of esculetin-treated mice were significantly decreased compared with those of the vehicle group on days 3, 5, and 7, respectively (Figure 1B). Esculetin also alleviated the weight loss in psoriasis-like mice on days 5, 6, and 7 (Figure 1C). We demonstrated that this dosage of esculetin did not cause any toxic injury to kidneys and livers of the mice 7 days after the treatment, as evidenced by H&E staining (Supplementary Figure S1). Taken together, our data suggest that esculetin ameliorates the skin lesion of IMQ-induced psoriatic mice.

### Esculetin inhibits CD3^+^ and CD8^+^ T cell infiltration in skin of psoriatic mice

Psoriasis is an autoimmune disease mainly mediated by CD3^+^ T cells. Our immunohistochemical results revealed that CD3 expression in the skin of vehicle group mice was significantly higher than that of control group (Figures 1D,F). Importantly, a significant decrease in CD3 expression was observed in mice treated with either low-dose of esculetin (ES-L) or high-dose of esculetin (ES-H) compared to the mice of vehicle group (*p* < 0.01 in both ES-L and ES-H groups vs. vehicle group, Figures 1D,F). Moreover, CD8+ T cells play an important role in the skin lesion in psoriasis (32–34). We found that esculetin, at a low- or high-dose, also suppressed CD8 expression in the skin of psoriatic mice (Figures 1E,G). These results suggest that esculetin hinders CD3^+^ or CD8^+^ T cell infiltration in the psoriatic skin.

### Esculetin inhibits epidermal hyperplasia in skin of psoriatic mice

**Figure 2:**
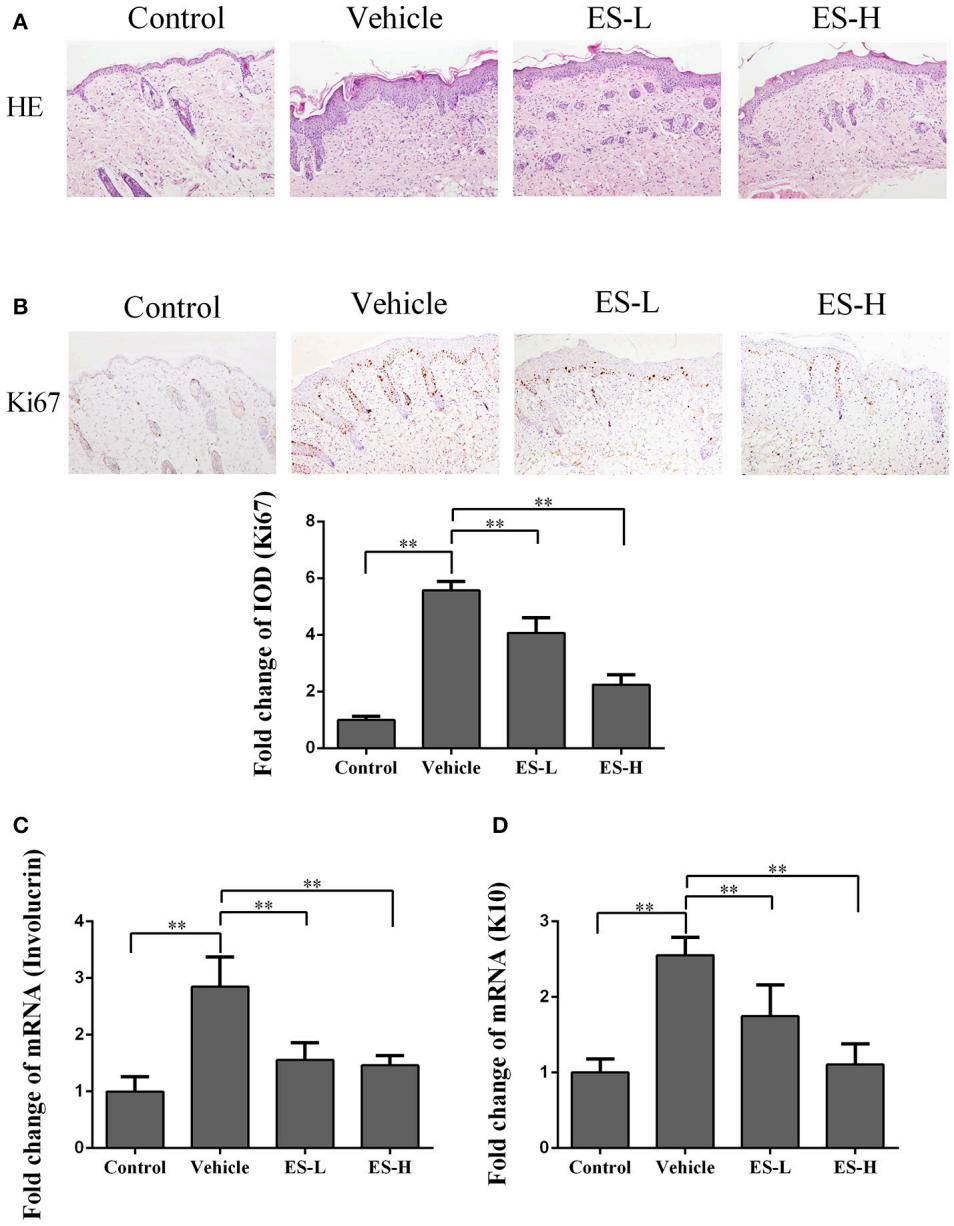
Esculetin inhibits epidermal hyperplasia in the skin of IMQ-induced psoriasis-like mice. H&E **(A)** and Ki67 **(B)** staining of the skin of control or IMQ-induced psoriasis-like mice 7 days after treatment without or with esculetin. Representative images of skin section are presented (magnification: 100X). The integrated optical density (IOD) of Ki67^+^ cells in the skin was calculated using ImagePro Plus. **(C,D)** qRT-PCR analysis of epidermal keratinocyte differentiation markers (Involucrin and Keratin 10) in the skin. Values were expressed as fold changes relative to control group that was set as 1.0. All data are presented as the mean ± SD (*n* = 4–5 mice/group, ** *p* < 0.01). One representative of three separate experiments is shown.

Psoriatic lesions are mainly characterized by hyperkeratosis and epidermal hyperplasia. Based on H&E staining, vehicle group mice exhibited significant epidermal hyperplasia, acanthosis, and hyperkeratosis in the epidermis 7 days after IMQ treatment. However, the epidermal hyperplasia was significantly alleviated when mice were treated with esculetin, especially at a high-dose (Figure 2A).

Ki67 is a nucleoprotein involved in cell proliferation. As shown in Figure 2B, immunohistochemical staining demonstrated that Ki67 expression was significantly increased in IMQ-treated mice when compared with control mice (5.57 vs. 1.0, *p* < 0.01). However, treatment with esculetin decreased Ki67 expression compared with vehicle group of IMQ-treated mice (*p* < 0.01 in both low-dose and high-dose of esculetin groups).

Involucrin is a differentiation marker of keratinocytes and mainly expressed in the cytoplasm. Quantitative RT-PCR analyses revealed that involucrin mRNA expression in vehicle group was significantly higher than that in control group (2.85-fold vs. 1.0, *p* < 0.01, Figure 2C). However, compared with vehicle group, esculetin treatment (both ES-L and ES-H) significantly reduced involucrin mRNA expression (both *p* < 0.01, Figure 2C). On the other hand, keratin 10 (K10) is a key protein associated with terminal differentiation of keratinocytes. K10 mRNA expression was increased in IMQ-induced mice when compared with control group (2.55-fold vs. 1.0, *p* < 0.01, Figure 2D). Similarly, esculetin lowered K10 mRNA expression compared with vehicle group (both *p* < 0.01, Figure 2D). These findings indicate that esculetin inhibits the proliferation and differentiation of keratinocytes in psoriatic mice.

### Esculetin lowers effector CD8^+^ T cell frequency in psoriatic mice

**Figure 3:**
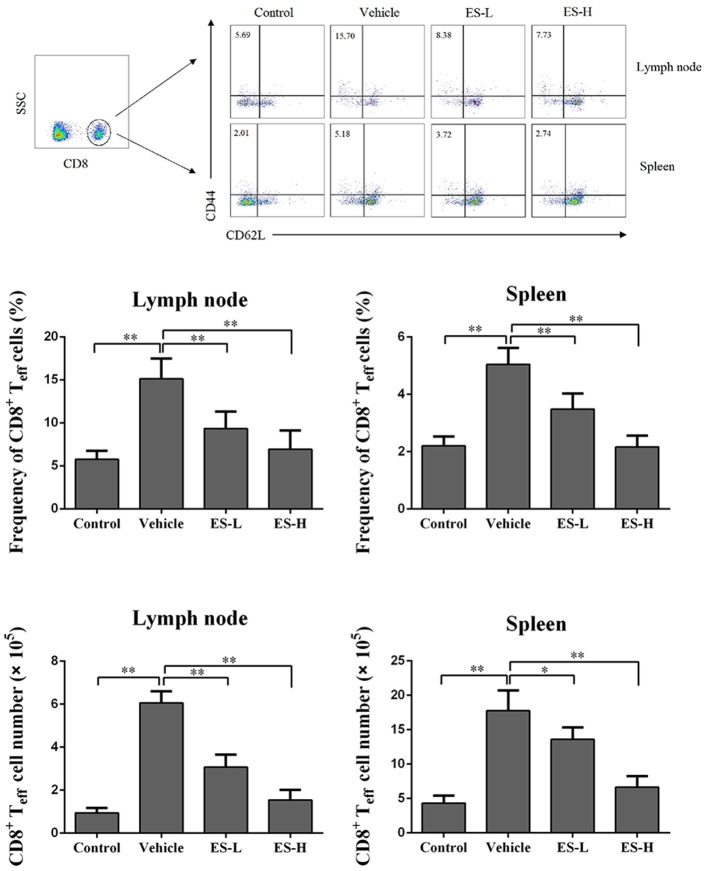
Esculetin decreases the frequency of CD8^+^CD44^high^CD62L^low^ effector T cells (CD8^+^ T_eff_) in IMQ-induced psoriasis-like mice. Draining lymph node and spleen cells were isolated from control or IMQ-induced psoriasis-like mice 7 days after treatment without or with esculetin. The frequencies and absolute numbers of CD8^+^CD44^high^CD62L^low^ in lymph nodes and spleens of the mice were determined *via* flow cytometric analysis. Data of column graphs are presented as the mean ± SD (*n* = 3–5, *p < 0.05 and **p < 0.01). One representative of three separate experiments is shown.

To evaluate whether esculetin would suppress effector CD8^+^ T cells (T_eff_), draining lymph node and spleen cells were isolated from IMQ-induced psoriasis-like mice 7 days after treatment without or with esculetin, and then analyzed using a flow cytometer. As shown in Figure 3, IMQ-treatment significantly increased the percentage of CD8^+^CD44^high^CD62L^low^ effector T cells (T_eff_) in both lymph nodes and spleens of the mice compared with control group (15.13 vs. 5.76 in lymph nodes and 5.05 vs. 2.21 in spleens, both *p* < 0.01). However, esculetin significantly decreased CD8^+^ T_eff_ percentages in both draining lymph nodes and spleens of psoriasis-like mice compared with vehicle group (Lymph node: mean = 9.34 at low-dose and 6.94 at high-dose vs. 15.13; and Spleen: mean = 3.49 at low-dose and 2.17 at high-dose vs. 5.05, all *p* < 0.01). Esculetin also significantly reduced the absolute numbers of the CD8^+^ T_effs_ in psoriatic mice (Figure 3). These findings suggest that esculetin hinders the development of CD8^+^CD44^high^CD62L^low^ effector T cells in psoriatic mice.

### Esculetin promotes the generation of CD4^+^Foxp3^+^ tregs *in vivo* and *in vitro*

**Figure 4:**
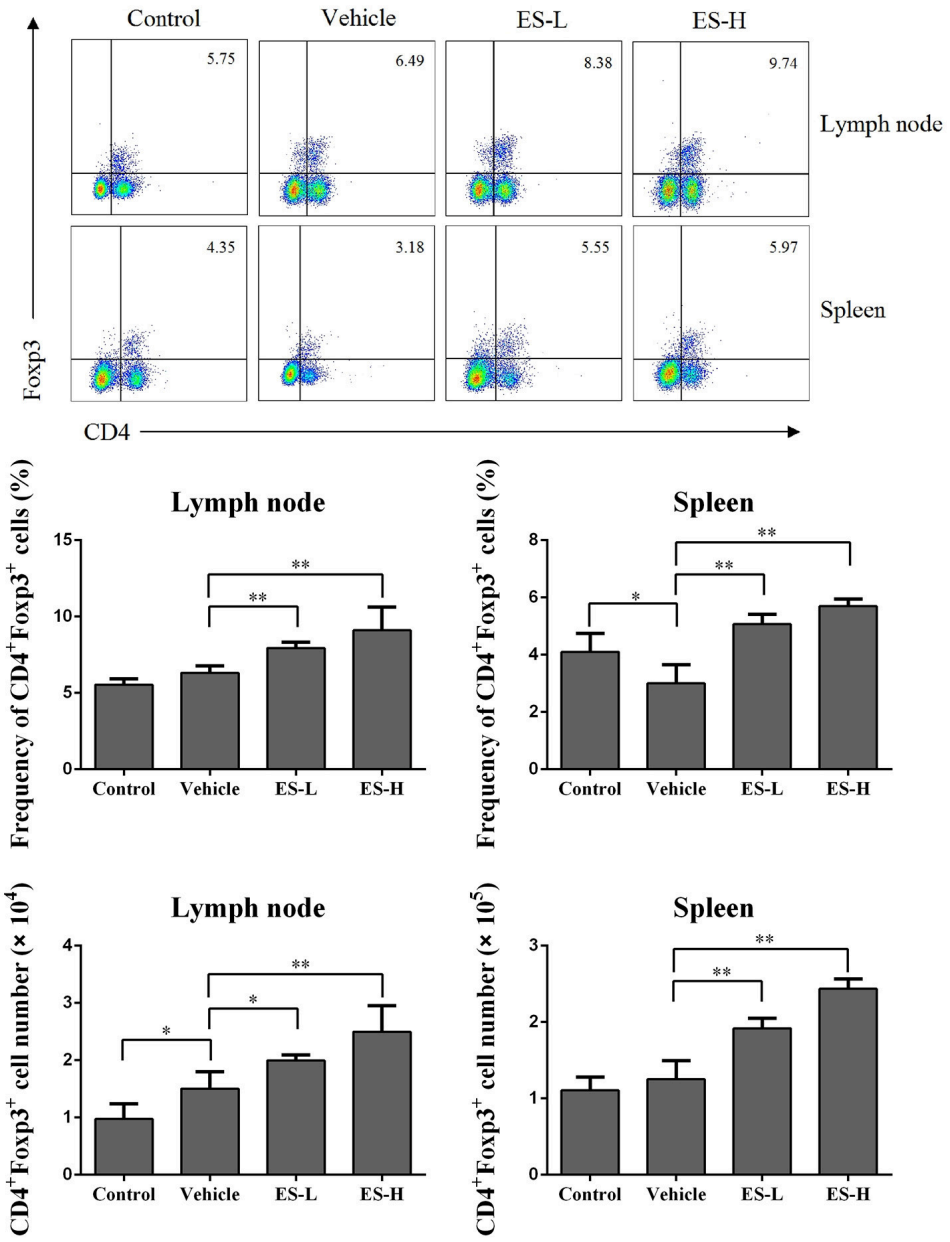
Esculetin ameliorates the skin lesion of IMQ-induced psoriasis-like mice by inducing CD4^+^Foxp3^+^ Tregs. Draining lymph node and spleen cells were isolated from IMQ-induced psoriasis-like mice 7 days after treatment without or with esculetin. The percentages and absolute numbers of CD4^+^Foxp3^+^ Tregs were measured via FACS analysis. Data of column graphs are presented as mean ± SD (*n* = 4–5, *p < 0.05 and **p < 0.01). One representative of three separate experiments is shown.

Regulatory T cells (Treg) play a critical role in preventing autoimmune diseases. Thus, we evaluated whether esculetin would alleviate psoriasis through inducing CD4^+^Foxp3^+^ Tregs *in vivo*. Draining lymph node and spleen cells were isolated from IMQ-induced psoriasis-like mice 7 days after treatment without or with esculetin, and CD4^+^Foxp3^+^ Tregs were detected via flow cytometric analysis. As shown in Figure 4, IMQ treatment in vehicle group slightly reduced the percentages of CD4^+^Foxp3^+^ Tregs in spleens, but not lymph nodes, of mice compared with control group. Importantly, esculetin significantly increased CD4^+^Foxp3^+^ Treg frequency in both draining lymph nodes and spleens of psoriasis-like mice compared with vehicle group (Lymph node: 8.25 vs. 6.32 in low-dose, and 9.61 vs. 6.32 in high-dose, both *p* < 0.01; Spleen: 5.07 in low-dose and 5.80 in high-dose vs. 3.01, both *p* < 0.01). Esculetin also significantly augmented the absolute numbers of the Tregs in psoriatic mice (Figure 4). However, esculetin treatment for 1 week did not alter the percentages and absolute numbers of the Tregs in mice that were not treated with IMQ (Supplementary Figure S2). In addition, esculetin significantly increased mRNA expression of FoxP3 in the skin compared with vehicle group (2.5 in low-dose vs. 1.3, *p* < 0.05 and 2.9 in high-dose vs. 1.3, *p* < 0.01, **Figure 7H**). These data suggest that esculetin promotes CD4^+^Foxp3^+^ Treg development *in vivo*.

**Figure 5:**
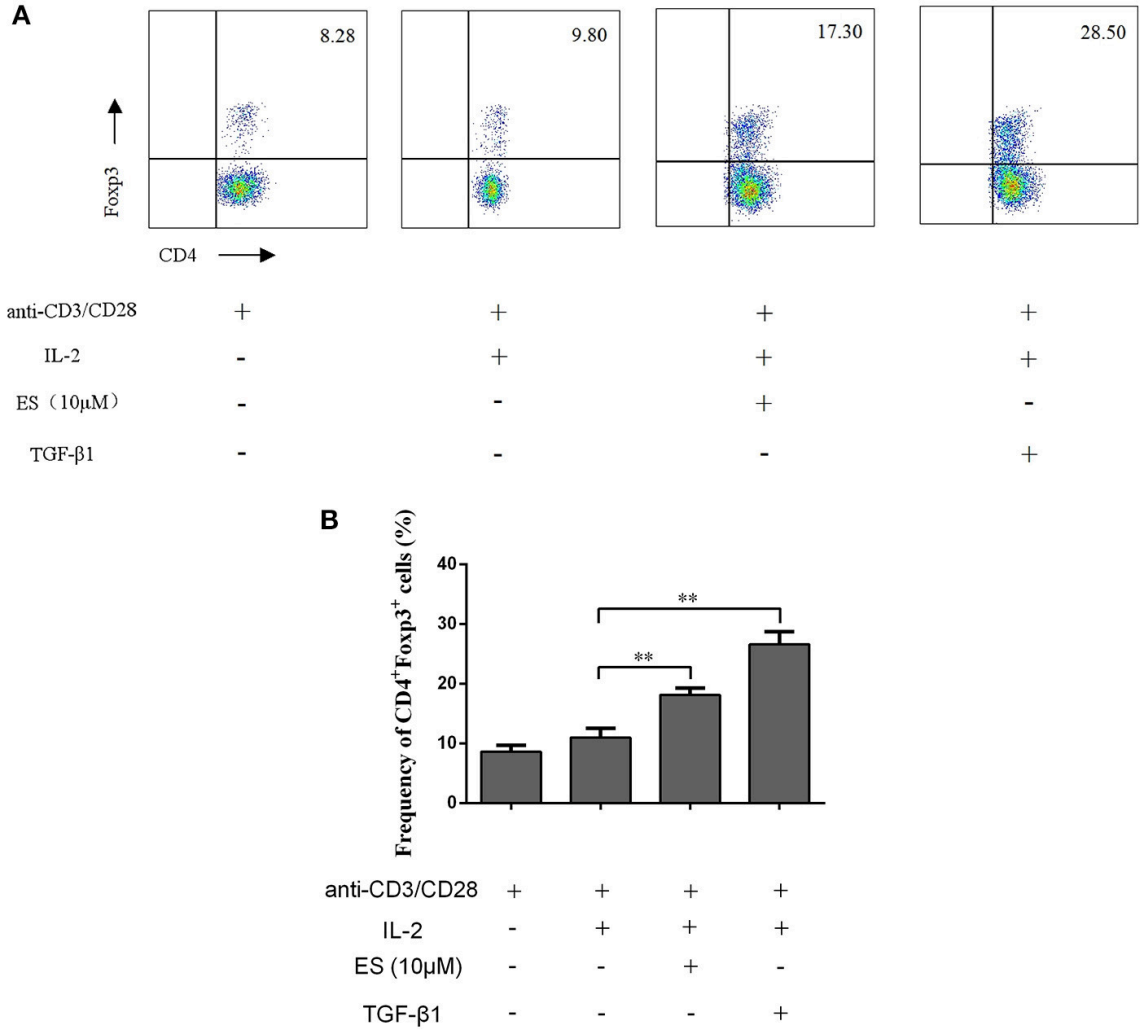
Esculetin also induces CD4^+^Foxp3^+^ Tregs *in vitro*. CD4^+^CD25^−^ T cells were isolated from naïve BALB/c mice *via* cell sorting by FACSAria III and then co-stimulated with anti-CD3 (5 μg/ml) plus anti-CD28 mAb (2.5 μg/ml) in the presence or absence of IL-2 (10 ng/ml) plus esculetin (10 μM) or TGF-β1 (5 ng/ml) for 4 days. CD4^+^Foxp3^+^ Treg frequencies were determined by flow cytometric analysis **(A)**. Data of column graphs are presented as the mean ± SD (*n* = 4–6, *p < 0.05 and **p < 0.01). One representative of three separate experiments is shown **(B)**.

To determine if esculetin could also induce CD4^+^Foxp3^+^ Tregs *in vitro*, CD4^+^CD25^−^ T cells were purified from naïve BALB/c mice via FACSAria cell sorting and then co-stimulated with anti-CD3 plus anti-CD28 mAb in the presence or absence of IL-2 plus esculetin or TGF-β1 for 4 days. CD4^+^Foxp3^+^ Tregs were enumerated by flow analysis. We found that esculetin significantly increased the frequency of CD4^+^Foxp3^+^ Tregs (Mean = 18.15 vs. 10.90, *p* < 0.01, Figure 5A,B). These data suggest that esculetin promotes the generation of CD4^+^Foxp3^+^ Tregs *in vitro*.

### Depletion of CD4^+^CD25^+^Foxp3^+^ tregs largely reverses esculetin-mediated reduction in PASI scores of psoriatic mice

**Figure 6:**
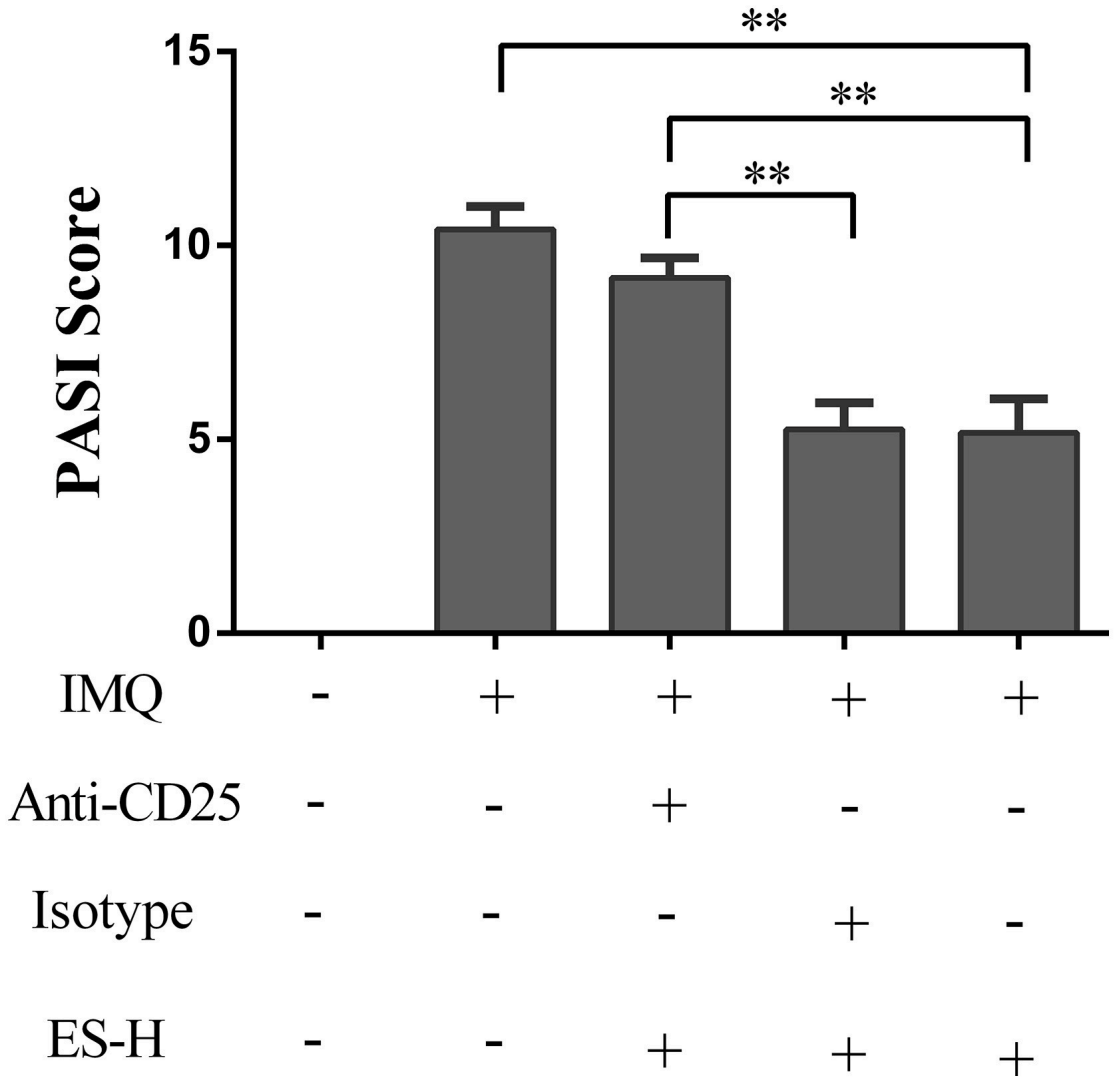
Depleting CD4^+^CD25^+^Foxp3^+^ Tregs largely reversed esculetin-mediated reduction in the PASI score of psoriatic mice. Shown are the PASI scores of the skin lesion in IMQ-induced psoriasis-like mice 7 days after treatment without or with high-dose of esculetin (ES-H) plus anti-CD25 Ab (PC61) or isotype control Ab. Data are presented as the mean ± SD (*n* = 4–5, **p < 0.01). One representative of three separate experiments is shown.

Given that esculetin induced CD4^+^Foxp3^+^ Tregs in IMQ-induced psoriasis-like mice, we further asked whether the effects of esculetin on skin lesion of the psoriatic mice were dependent on CD4^+^Foxp3^+^ Tregs. BALB/c mice were topically administered with IMQ and treated with high-doses of esculetin (ES-H). CD4^+^CD25^+^Foxp3^+^ Tregs in the mice were then depleted by anti-CD25 mAb (PC61). As shown in Figure 6, depleting CD4^+^Foxp3^+^ Tregs mostly reversed esculetin-mediated reduction in the PASI score while isotype control Ab did not alter the PASI score. Our results indicate that antipsoriatic effects of esculetin are largely dependent on CD4^+^Foxp3^+^ Tregs.

### Esculetin suppresses mRNA expression of proinflammatory cytokines in IMQ-induced psoriatic mice

**Figure 7:**
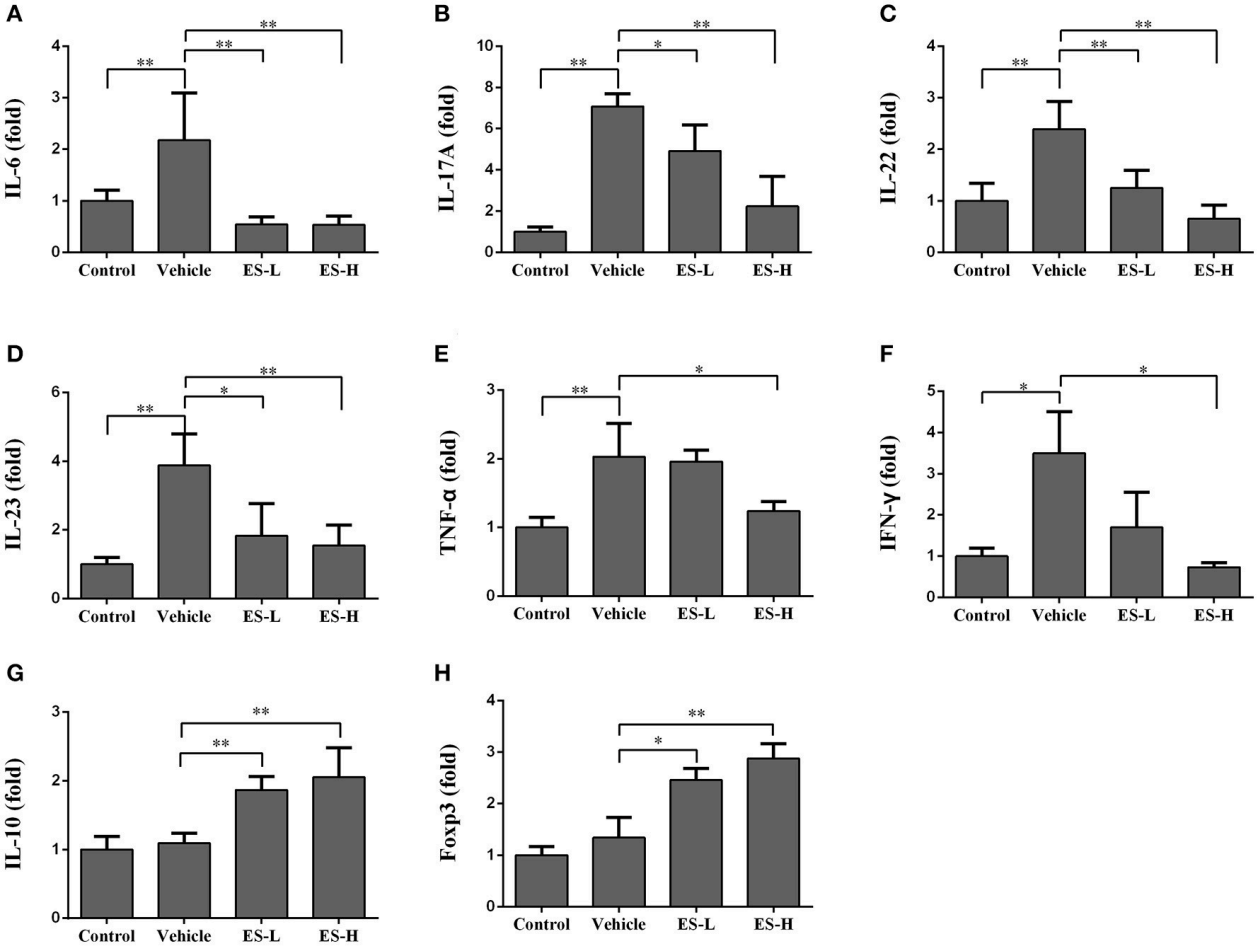
Esculetin inhibits the mRNA expression of proinflammatory cytokines while increasing Treg-related cytokine expression in psoriatic mouse skin. The mRNA levels of IL-6 **(A)**, IL-17A **(B)**, IL-22 **(C)**, IL-23 **(D)**, TNF-α **(E)**, IFN-γ **(F)**, IL-10 **(G)**, and Foxp3 **(H)** in the skin of IMQ-induced psoriasis-like mice were determined by RT-PCR analysis 7 days after the treatment without or with esculetin. Values were expressed as fold changes relative to control group that was set as 1.0. Data are presented as the mean ± SD (*n* = 4–6, *p < 0.05 and **p < 0.01). One representative of three separate experiments is shown.

We further examined the effects of esculetin on mRNA expression of proinflammatory cytokines in the skin of IMQ-induced psoriatic mice using RT-PCR 7 days after treatment without or with esculetin. As shown in Figures 7A–F, the mRNA levels of IL-6, TNF-α, IFN-γ, and Th17 cytokines (IL-17A, IL-22, IL-23) in vehicle group were significantly higher than those in control group (*p* < 0.05 or *p* < 0.01 as indicated). Administration of esculetin, especially at high-doses, significantly reduced the mRNA expressions of these proinflammatory cytokines compared with vehicle group of IMQ-treated mice (*p* < 0.05 or *p* < 0.01). However, esculetin treatment significantly increased the mRNA levels of IL-10 (Figure 7G) and FoxP3 (Figure 7H) in the skin compared to vehicle group without esculetin treatment.

### Esculetin inhibits NF-κB signaling in psoriatic skin

**Figure 8:**
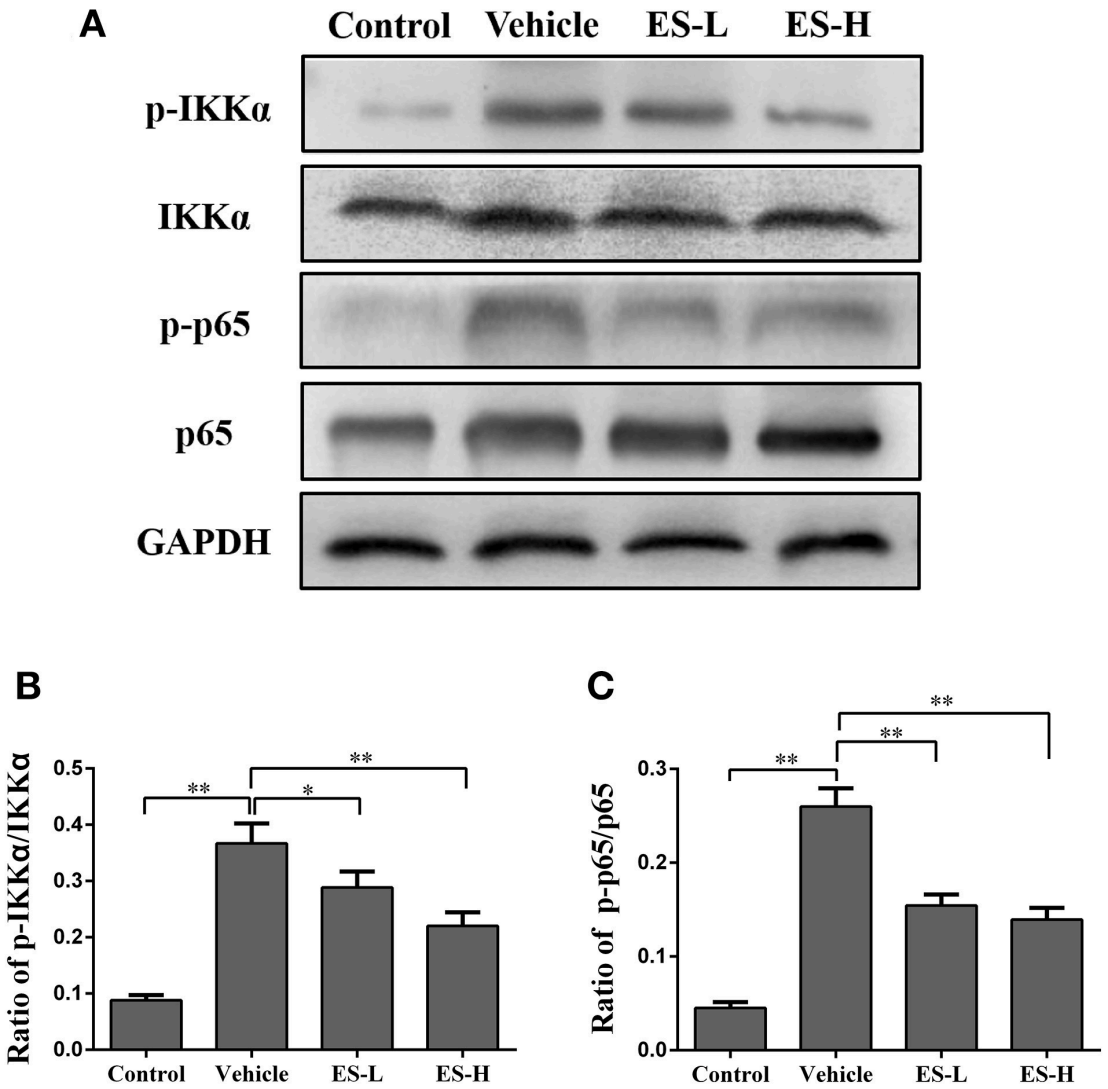
Esculetin inhibits NF-κB signaling in the skin of IMQ-induced psoriasis-like mice. **(A)** A representative of Western blot images of p-IKKα, IKKα, p-p65, and p65 expressions in the skin of IMQ-induced psoriasis-like mice 7 days after the treatment without or with esculetin. The densitometry analyses of the Western blotting images are shown as the relative expression of p-IKKα/IKKα **(B)** and p-p65/p65 **(C)**. GAPDH was used as an internal control. Values are expressed as ratios of p-IKKα/IKKα or p-p65/p65. Data are presented as the mean ± SD from four independent experiments (*n* = 4, *p < 0.05 and **p < 0.01).

Since NF-κB signaling is closely associated with the release of proinflammatory cytokines in psoriasis, we determined whether esculetin would have an impact on NF-κB signaling in IMQ-induced psoriatic mice. The expressions of phosphorylated IKKα (phospho-IKKα), IKKα, phospho-p65 and p65 proteins in the mouse skin were measured by western blotting. As shown in Figure 8A, we found that phospho-IKKα and phospho-p65 expressions were significantly increased when mice were treated with IMQ (both *p* < 0.01). Importantly, esculetin effectively inhibited the expression of phospho-IKKα (low-dose: *p* < 0.05 and high-dose: *p* < 0.01, Figure 8B) and phospho-p65 (both low-dose and high-dose: *p* < 0.01, Figure 8C) compared with the vehicle group. These results indicate that esculetin exerts its antipsoriatic effects through blocking NF-κB signaling pathway.

## Discussion

Psoriasis is an inflammatory skin disease, which is characterized by epidermal hyperplasia, immune cell infiltration, and parakeratosis. Although the exact pathogenesis of psoriasis is not fully understood, it is widely accepted that NF-κB signaling pathway plays an important role in the pathogenesis of psoriasis (35, 36). NF-κB is a key factor in immune-based inflammatory diseases, including psoriasis, and closely related to numerous cellular and pathological processes, such as keratinocyte proliferation, and differentiation (37). Thus, inhibition of NF-κB activation would help treat psoriasis (38). It has been reported that some natural products derived from herbal medicine are effective in treating psoriasis by suppressing dendritic cell activation or regulating Th17/Treg balance (39–43). Esculetin is an active derivative of the coumarin and can be isolated from the bark of some natural plants, including *Fraxinus rhynchophylla* Hance, *Fraxinus chinensis* Roxb, and *Fraxinus stylosa* Lingelsh (14, 15). Previous studies have demonstrated that esculetin exerts its anti-inflammatory effects by inhibition of NF-κB activation (27, 28). In particular, esculetin can alleviate atopic dermatitis by downregulating NF-κB activation (25). However, it is unknown whether esculetin has an effect on psoriasis. Here we have provided the first evidence that esculetin ameliorates murine psoriasis via suppression of NF-κB signaling.

To investigate the effects of esculetin on the epidermal barrier, we measured the markers for proliferation and differentiation of keratinocytes in the epidermis. Ki-67 is a nuclear protein that is strictly associated with cell proliferation (44) and expressed in the basal keratinocyte layers of the epidermis. The keratinocyte proliferation was found in IMQ-induced psoriatic mice and psoriatic patients (45). Previous studies also demonstrated that the numbers of epidermal Ki-67^+^ cells were increased in psoriatic skin (46). In present study, we found that large numbers of Ki-67^+^ cells were present in the basal layer of the epidermis after mice were treated with IMQ while Ki-67^+^ cells were absent in the epidermis of normal mice. Importantly, we found that esculetin decreased Ki-67^+^ expression, suggesting that it has an impact on abnormal proliferation of keratinocytes. On the other hand, involucrin is a protein component of human skin and is mainly expressed in the granular and upper-spinous layers in normal skin (47, 48). It provides structural support to cells, allowing cells to resist invasion (49, 50). It was reported that involucrin extended into the middle spinous layer in psoriatic patients (46). An increase in involucrin expression was also observed in mice treated with IMQ (51). Indeed, our qRT-PCR results revealed that IMQ increased involucrin mRNA expression. However, esculetin reduced its mRNA expression compared to IMQ alone, implying that esculetin has an inhibitory effect on keratinocytes.

Imiquimod (IMQ), a ligand for Toll-like receptor 7/8 and immune activator, has been widely used to establish a psoriasis-like mouse model (52, 53). It has been proved that IMQ-induced psoriasis-like skin inflammation is a typical disease associated with IL-23/IL-17 axis imbalance (54, 55). Upregulated IL-23 and IL-17 have been found in both psoriatic patients (56) and psoriasis-like mice (57). IL-23 activates Th17 cells through STAT3 pathway, thus producing Th17-related cytokines, such as IL-17, IL-22, and TNF-α (58). Thus, proinflammatory cytokines, such as TNF-α and IL-17, are considered the therapeutic targets for the treatment of psoriasis (41, 59). In addition, IL-6 protein has been shown to increase in psoriatic skin. Targeting IL-6 signaling in psoriasis may enhance immunosuppression by Tregs (60). Studies have also revealed that IFN-γ is also highly expressed in psoriasis (61). These cytokines in turn can activate keratinocytes. We found that esculetin reduced mRNA levels of IL-6, IL-23, IL-22, IL-17A, IFN-γ, and TNF-α in psoriatic mouse skin, suggesting that esculetin may ameliorate the skin inflammation of psoriatic mice via inhibiting Th1 and Th17 cell differentiation.

The imbalance of Th17/ Treg plays a critical role in the development of psoriasis (62, 63). CD4^+^CD25^+^Foxp3^+^ Tregs can differentiate into IL-17A-producing Th17 cells in patients with psoriasis (64). Previous studies have shown that induction of endogenous CD4^+^CD25^+^Foxp3^+^ Tregs or reversal of Th17/Treg imbalance ameliorates IMQ-induced psoriasis in mice (65, 66). CD4^+^Foxp3^+^ Tregs could circulate through the lymphoid tissues, such as draining lymph nodes, resulting in suppression of effector T cell activation and immune responses in skin (67). In our study, we found that esculetin upregulated CD4^+^Foxp3^+^ Treg frequency but downregulated effector T cell frequency in psoriatic mice. It also increased the mRNA level of FoxP3 in the lesion skin of psoriatic mice while promoting the differentiation of naïve CD4^+^CD25^−^ T cells into CD4^+^Foxp3^+^ Tregs *in vitro*. Depletion of CD4^+^Foxp3^+^ Tregs largely reversed the esculetin-mediated reduction in PASI scores of psoriatic mice, suggesting that esculetin exerts its anti-psoriatic effects largely through induction of CD4^+^Foxp3^+^ Tregs. Interestingly, it has been reported that cyclosporine, a commonly used immunosuppressant in clinic, hinders tolerance induction when combined with costimulatory blockade-based regimen (68–70) because it can block IL-2 expression (71–73), which is essential to Treg function (74–76). Thus, as a new immunosuppressant, esculetin may have an advantage over cyclosporine since the former does not inhibit IL-2 expression (26).

We demonstrated that esculetin induced Tregs *in vivo* and *in vitro*. Previous studies have revealed that NF-κB is critical for regulating Treg cell development (77, 78). Schuster et al found that Tregs were induced by inhibition of NF-κB activation (79). Therefore, induction of Tregs by esculetin in our study could be attributed to its ability to suppress NF-κB activation. In addition, NF-κB activation reportedly was upregulated in IMQ-induced psoriatic mice (29) and psoriatic patients (30). On the other hand, NF-κB in keratinocytes can be activated by proinflammatory cytokines, resulting in the proliferation of epidermal keratinocytes in psoriasis (10). Therefore, we investigated whether esculetin would alleviate psoriasis via inhibition of NF-κB signaling. We found that esculetin inhibited the phosphorylation of p65 and IKKα, suggesting that esculetin ameliorates the skin lesion in IMQ-induced psoriatic mice by inhibiting NF-κB signaling.

The exact mechanisms underlying esculetin-mediated reduction in proinflammatory cytokines remain to be defined. The outcome of diminished proinflammatory cytokines is likely caused by an increase in Treg numbers and/or downregulation of NF-κB activity. Tregs suppress immune-based inflammatory reactions while NF-κB signaling mediates inflammation. Further studies are needed to determine if an increase in Treg numbers results in a decrease in NF-κB activity following esculetin treatment, or vice verse.

## Author contributions

YC, QZ, and HL performed experiments. CL contributed the project idea and vital reagents. C-LL, FQ, and LH analyzed the data. ZD designed experiments and edited the manuscript.

### Conflict of interest statement

The authors declare that the research was conducted in the absence of any commercial or financial relationships that could be construed as a potential conflict of interest.

## Abbreviations

DC: dendritic cell
IMQ: Imiquimod
PASI: Psoriasis Area and Severity Index
Treg: Regulatory T cell.
