# Supplementary figures and images for: Esculetin Ameliorates Psoriasis-Like Skin Disease in Mice by Inducing CD4+Foxp3+ Regulatory T Cells
Source: Front Immunol. 2018 Sep 12;9:2092. doi: 10.3389/fimmu.2018.02092 (PMC6143660; doi:10.3389/fimmu.2018.02092)

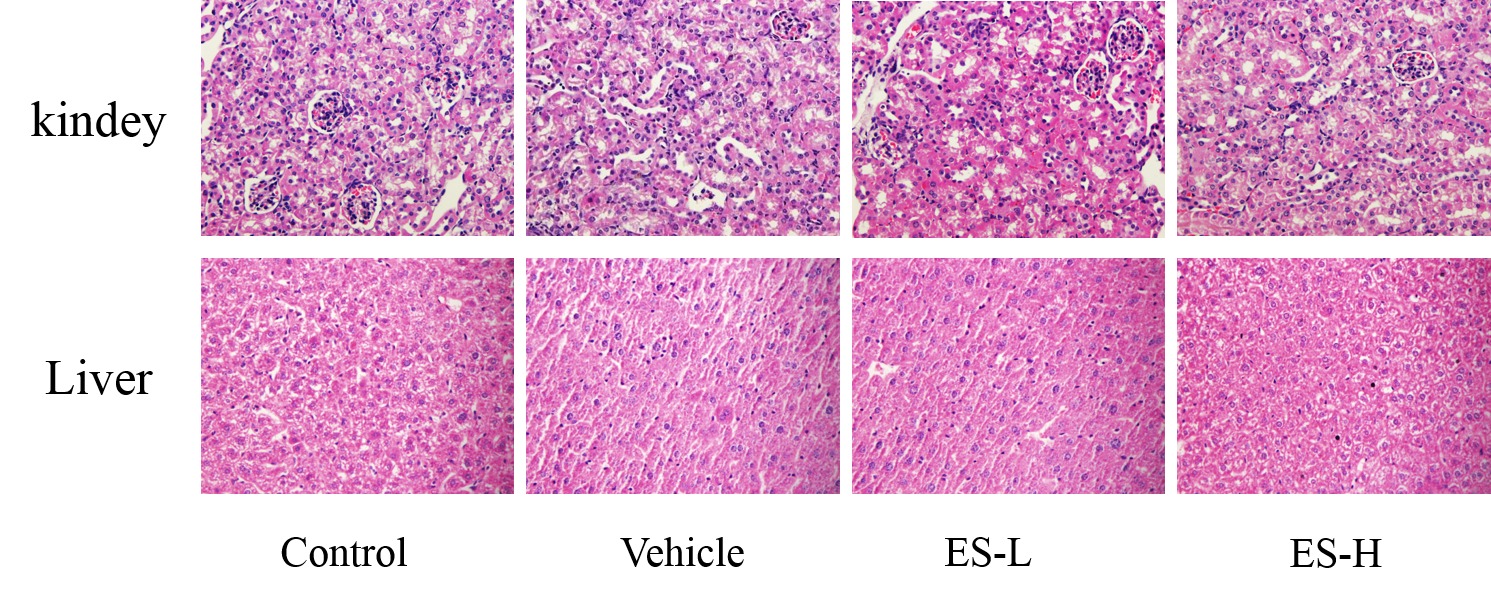

Supplement: Supplementary file 1 [file Image_1.TIF]

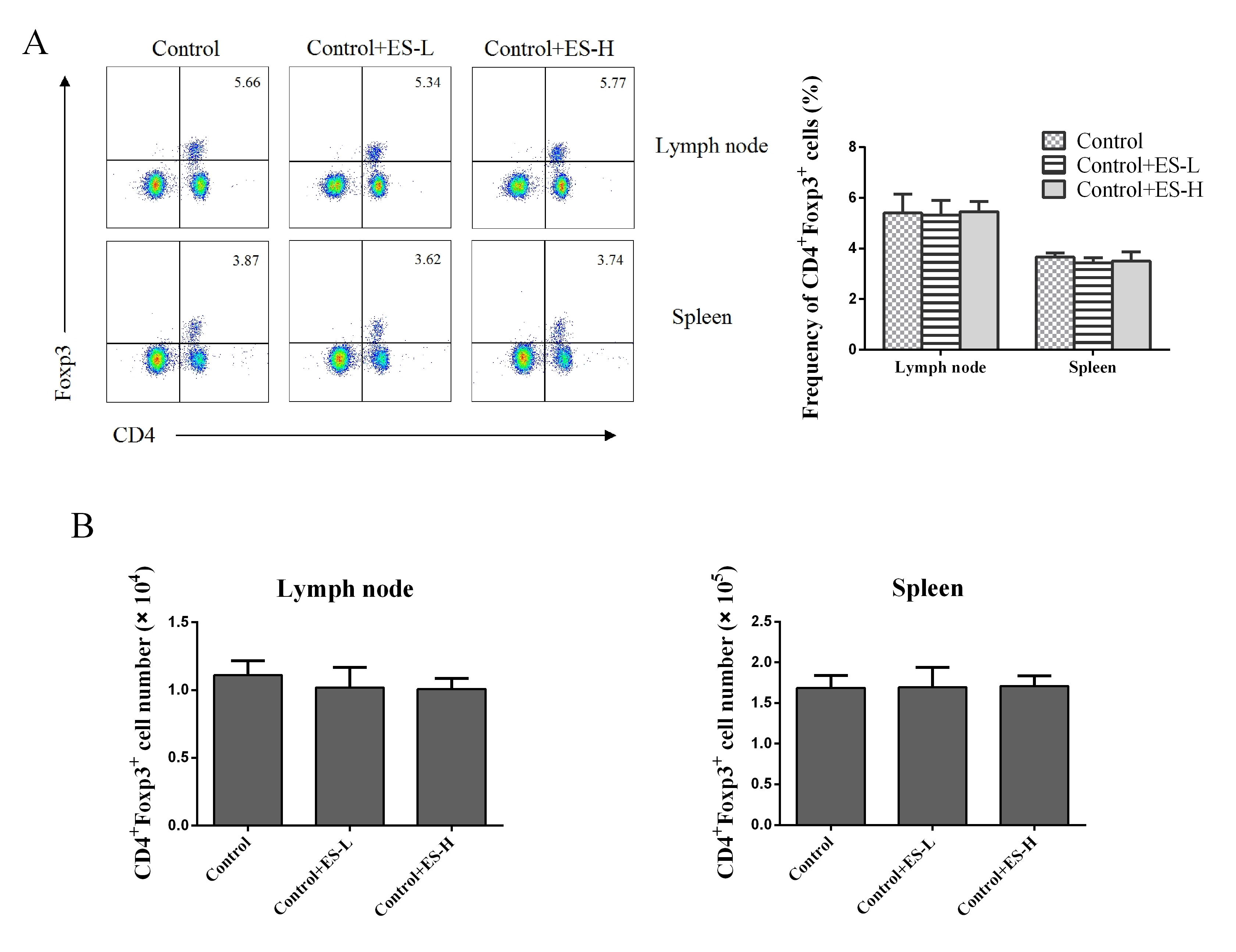

Supplement: Supplementary file 2 [file Image_2.JPEG]
